# Perivascular Adipose Tissue Inhibits Endothelial Function of Rat Aortas via Caveolin-1

**DOI:** 10.1371/journal.pone.0099947

**Published:** 2014-06-13

**Authors:** Michelle Hui-Hsin Lee, Shiu-Jen Chen, Cheng-Ming Tsao, Chin-Chen Wu

**Affiliations:** 1 Graduate Institute of Life Sciences, National Defense Medical Center, Taipei, Taiwan; 2 Department of Physiology, National Defense Medical Center, Taipei, Taiwan; 3 Department of Anesthesiology, National Defense Medical Center, Taipei, Taiwan; 4 Department of Pharmacology, National Defense Medical Center, Taipei, Taiwan; 5 Department of Nursing, Kang-Ning Junior College of Medical Care and Management, Taipei, Taiwan; 6 Department of Anesthesiology, Taipei Veterans General Hospital and National Yang-Ming University, Taipei, Taiwan; 7 Department of Pharmacology, Taipei Medical University, Taipei, Taiwan; University of Iowa, United States of America

## Abstract

Perivascular adipose tissue (PVAT)-derived factors have been proposed to play an important role in the pathogenesis of atherosclerosis. Caveolin-1 (Cav-1), occupying the calcium/calmodulin binding site of endothelial NO synthase (eNOS) and then inhibiting nitric oxide (NO) production, is also involved in the development of atherosclerosis. Thus, we investigated whether PVAT regulated vascular tone via Cav-1 and/or endothelial NO pathways. Isometric tension studies were carried out in isolated thoracic aortas from Wistar rats in the presence and absence of PVAT. Concentration-response curves of phenylephrine, acetylcholine, and sodium nitroprusside were illustrated to examine the vascular reactivity and endothelial function. The protein expressions of eNOS and Cav-1 were also examined in aortic homogenates. Our results demonstrated that PVAT significantly enhanced vasoconstriction and inhibited vasodilatation via endothelium-dependent mechanism. The aortic NO production was diminished after PVAT treatment, whereas protein expression and activity of eNOS were not significantly affected. In addition, Cav-1 protein expression was significantly increased in aortas with PVAT transfer. Furthermore, a caveolae depleter methyl-*β*-cyclodextrin abolished the effect of PVAT on the enhancement of vasoconstriction, and reversed the impairment of aortic NO production. In conclusion, unknown factor(s) released from PVAT may inhibit endothelial NO production and induce vasocontraction via an increase of Cav-1 protein expression.

## Introduction

Adipose tissue is divided into white and brown adipose tissues, which store excess energy and regulate body temperature, respectively [1], [2]. In addition, adipose tissue acts as a secretory organ to release several adipokines [3]. These adipokines include hormones (e.g. leptin and adiponectin), inflammatory cytokines (e.g. tumor necrosis factor-*α* (TNF-*α*), interleukin-6 (IL-6), omentin, and visfatin), and other proteins (e.g. plasminogen activator inhibitor-1, angiotensinogen, resistin, and apelin) [4], [5]. Most of blood vessels are directly surrounded by adipose tissue, known as perivascular adipose tissue (PVAT) except for cerebrovessels.

Initially, PVAT is considered as structural support for the vasculature, and it is usually removed in isometric vascular tone studies. In 1991, Soltis and Cassis demonstrate that PVAT attenuates vascular response of norepinephrine (NE) in isolated rat aorta preparations as a result of reuptaking NE by adrenergic nerves in PVAT [6]. However, Löhn and his colleagues show that the vascular responses to angiotensin II (Ang II), serotonin and phenylephrine (PE) are reduced in intact aortas with PVAT, not subject to the uptake by adrenergic nerves [7], suggesting that the anti-contractile effect of PVAT is most likely mediated by a transferable PVAT-released material, adventitium-derived relaxing factor (ADRF), which relaxes different kinds of arteries [7]. In contrast, Gao *et al*. disclose that PVAT enhances the contractile response of mesenteric artery to perivascular nerve stimulation through the production of superoxide [8]. Moreover, PVAT diminishes the vasodilating effect of acetylcholine (ACh) in coronary arteries of lean dogs via phosphorylation of endothelial nitric oxide (NO) synthase (eNOS) and inhibiting eNOS activity and NO production [9], [10]. In addition, the paracrine role of PVAT may involve in endothelial dysfunction leading to local stimulation of atherosclerotic plaque formation [11].

Caveolin-1 (Cav-1), a structure protein of caveolae, is present in most of the cells and involved in the development of atherosclerosis [12]. This 22-kDa protein-rich striation, Cav-1, inhibits endothelial function by occupying of the calcium/calmodulin (Ca^2+^/CaM) binding site of eNOS [13]. Caveolae are small flask-shaped invaginations at the plasma membrane enriched with cholesterol, glycosphingolipids and Cav, which interacts with a variety of signal-transducing molecules [13]. In addition, adipose tissue is a main site for cholesterol storage [14], and the crucial role of cholesterol in caveolae biogenesis has been evident for quite some time. However, whether PVAT could (i) regulate caveolae formation, (ii) increase the expression of Cav-1, or (iii) change vascular tension in rat aorta is unclear. Therefore, we tried to examine the role of PVAT in regulation of vascular tone via Cav-1 and/or endothelial NO by using wire myography and protein expression analysis.

## Materials and Methods

### Materials

Potassium chloride (KCl), PE, N^ω^-nitro-L-arginine methyl ester (L-NAME, a NOS inhibitor), ACh, sodium nitroprusside (SNP), 4-Hydroxy-TEMPO (tempol, a superoxide anion scavenger), indomethacin (a non-selective cyclooxygenase (COX) inhibitor), and methyl-*β*-cyclodextrin (CD, a cholesterol depleter which disassembles caveolae) were purchased from Sigma-Aldrich (St. Louis, MO, USA); losartan (an Ang II type 1 receptor antagonist) was from Cayman Chemical Company (Ann Arbor, MI, USA). Drug concentrations were expressed as final molar concentrations in the bath chamber. 10× radio-immunoprecipitation assay (RIPA) lysis buffer was from Millipore Corporate Headquarters (Temecula, CA, USA); bicinchoninic acid (BCA) protein assay kit and Pierce enhanced chemiluminescent (ECL) Western Blotting Substrate were from Thermo Fisher Scientific (Rockford, IL, USA); anti-p-eNOS^Thr495^ was from Santa Cruz Biotechnology (Santa Cruz, CA, USA); anti-p-eNOS^Ser1177^ was from Abcam plc (Cambridge, UK); anti-eNOS, anti-adenosine monophosphate-activated protein kinase (anti-AMPK), anti-Cav-1, anti-*β*-actin, and horseradish peroxidase (HRP) conjugated goat anti-mouse IgG were from BD Transduction Laboratorie (Lexington, KY, USA); HRP conjugated goat anti-rabbit IgG was from Cell Signaling Technology (Danvers, MA, USA).

### Ethics statements

All animals used in this study received humane care in compliance with the animal care guidelines of the institute. This study was performed in accordance with the criteria of the National Academy of Sciences, and approved by the Institutional Animal Care and Use Committee of National Defense Medical Center (Taipei, Taiwan).

### Animals

Adult male Wistar rats (250 to 300 g) were provided by BioLasco Taiwan Co., Ltd. (Taipei, Taiwan) and raised in the Laboratory Animal Center of National Defense Medical Center (Taipei, Taiwan). The animals were maintained on a 12-hour light/dark cycle and were given free access to water and standard rat chow.

### Preparation of isolated thoracic aortic rings

**Figure 1:**
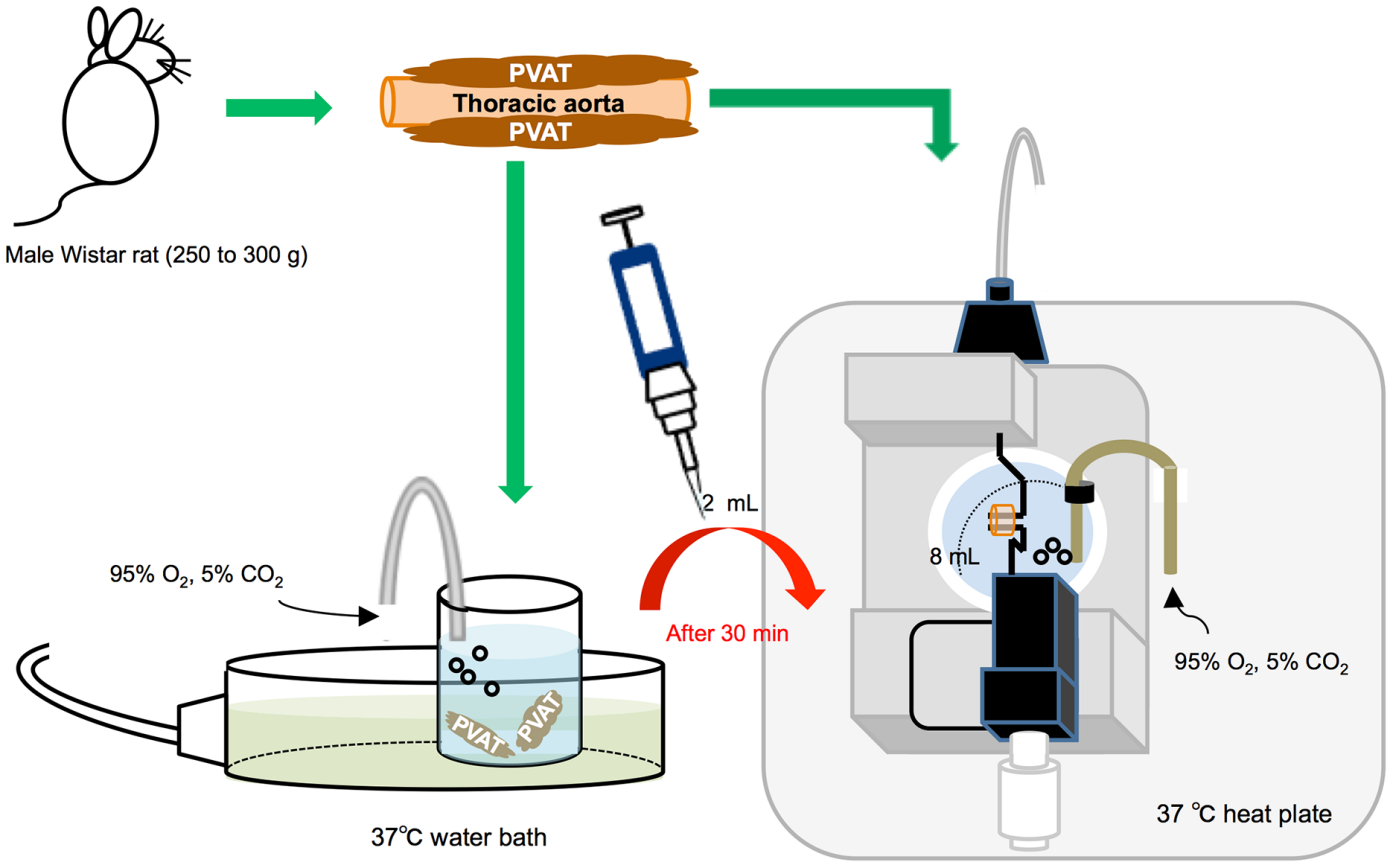
A simple scheme of vascular reactivity protocol. After all perivascular adipose tissue (PVAT) was excised, the isolated PVAT was incubated in another bath chamber with Krebs' solution for 30 min before the isometric tension studies. At the beginning of experiment, we replaced 2 mL of the PVAT-incubated solution in a donor bath chamber to the aorta in an acceptor chamber. In some experiments, the endothelium was removed.

Rats were euthanatized by an intraperitoneal injection of sodium pentobarbital (80 mg/kg). After rats were sacrificed, thoracic aortas were rapidly isolated and transferred to cold (4°C) and oxygenated (95% O_2_ and 5% CO_2_) Krebs' solution containing (in mM, pH 7.4): 118 NaCl, 25 NaHCO_3_, 11 glucose, 4.7 KCl, 1.17 MgSO_4_, 1.2 KH_2_PO_4_, 2.5 CaCl_2_. Then, all PVAT surrounded aorta was excised (Fig. 1). In some experiments, the endothelium was removed (i.e. (-E) group) by gently rubbing the intimal surface.

### Vascular reactivity in the thoracic aortas

**Figure 2:**
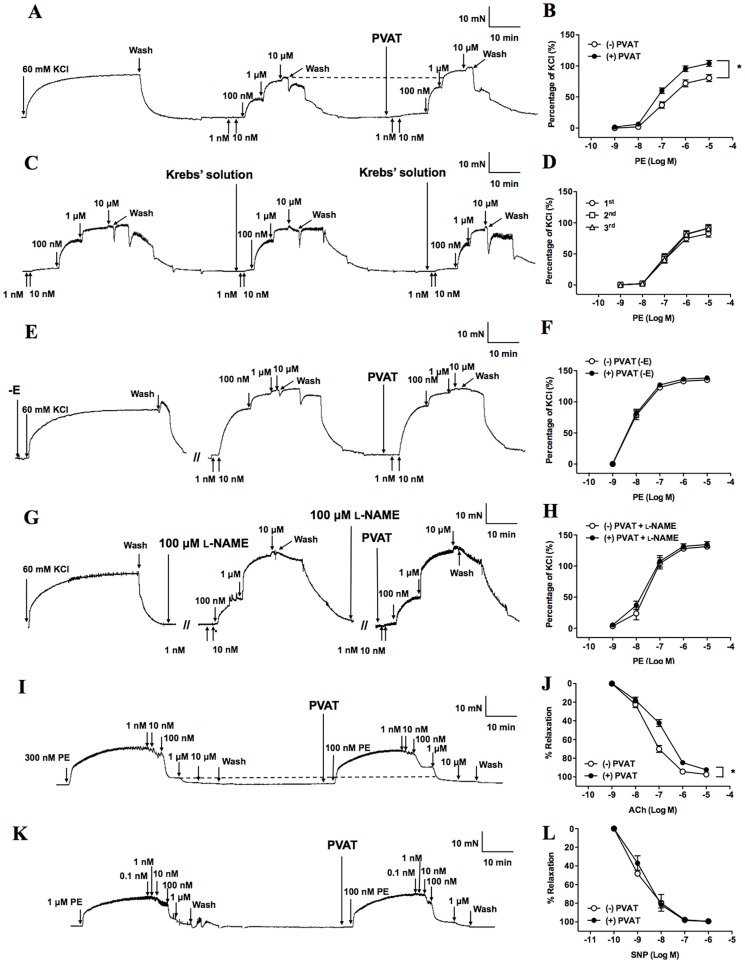
The association between endothelium, endothelial vasodilators and perivascular adipose tissue (PVAT). Original traces demonstrate contractile responses to incremental concentrations of phenylephrine (PE) (1 nM∼10 µM) in the presence and absence of PVAT (A).Statistical analysis of the PE-induced vasocontraction in the presence and absence of PVAT (n = 8) (B). Control experiment was shown as both original trace (C) and statistical analysis (n = 13) (D) of three times repeated contractile response to incremental concentrations of PE (1 nM∼10 µM). After endothelium denuded (-E) (E) or pretreatment of aorta with N^ω^-nitro-L-arginine methyl ester (L-NAME, 100 µM) (G), the original traces demonstrate contractile responses to incremental concentrations of PE (1 nM∼10 µM) in the presence and absence of PVAT. -E (n = 5) (F) or pretreatment of aorta with L-NAME (100 µM, n = 7) (H), concentration-response curves to PE was examined in the presence and absence of PVAT. Original traces demonstrate dilator response to incremental concentrations of acetylcholine (ACh) (1 nM∼10 µM) (I) or sodium nitroprusside (SNP) (0.1 nM∼1 µM) (K) in the presence and absence of PVAT. Vasodilator responses to ACh (n = 24) (J) and SNP (n = 5) (L) were performed in aortas in the presence and absence of PVAT. Values are means ± SEM for the numbers of animals in parentheses. *P<0.05 by two-way ANOVA compared with (−) PVAT of same treatment group. KCl, potassium chloride; (−) PVAT, absence of PVAT; (+) PVAT, presence of PVAT; 1^st^, PE-induced vasocontraction at the first time; 2^nd^, PE-induced vasocontraction at the second time; 3^rd^, PE-induced vasocontraction at the third time.

Segments of thoracic aorta (2 mm) were mounted in bath chambers of the wire myograph (8 mL) (Multi Wire Myograph System-Model 620 M, Danish Myo Technology, Aarhus, Denmark) in Krebs' solution at 37°C, continuously bubbled with 95% O_2_ and 5% CO_2,_ in order to measure the isometric tension (Fig. 1). The aortas were allowed to equilibrate under zero tension for 15 min to be stretched to their optimal lumen diameter for active tension development. The basic tension was determined based on the internal circumference (IC)/wall tension ratio of the segments by setting the IC_0_ to 90% of what the vessels would have if they were exposed to a passive tension equivalent to that produced by a transmural pressure of 100 mmHg (IC_100_). The diameter (I_1_) was determined according to the equation I_1_ = IC_1_/π [15], using specific software for normalization (DMT Normalization Module, LabChart 7, ADInstruments, New Zealand). In addition, the isolated PVAT was incubated in another bath chamber with Krebs' solution same as the above condition for 30 min before the isometric tension studies. After equilibration for 1 h, the intactness of endothelium was confirmed by ACh (1 µM) in PE (1 µM)-precontracted vessels. More than 90% vasorelaxation induced by ACh was considered as no injury of the endothelium. Vascular contraction was assessed by the contractile response to PE (1 nM to 10 µM), and vascular tension was expressed as a percentage of the steady-state tension (100%) induced by external 60 mM KCl. Endothelium-dependent and -independent relaxations in PE (20 nM∼1 µM)-precontracted vessels were assessed by measuring the dilatory response to ACh (1 nM to 10 µM) and SNP (0.1 nM to 1 µM), respectively. The vascular dilatation was expressed as a percentage of maximum response to PE (20 nM∼1 µM). In some experiments, we replaced 2 mL of the PVAT-incubated solution in a donor bath chamber to the aorta in an acceptor chamber (Fig. 1). In control experiments, transfer of Krebs' solution in a donor bath chamber without PVAT incubation did not affect the tension of aorta in an acceptor chamber (Fig. 2C and 2D). L-NAME (1, 3, 10, or 100 µM, 30 min), tempol (100 µM, 15 min), losartan (10 µM, 30 min), indomethacin (10 µM, 30 min) and CD (100 µM or 1 mM, 30 min) were incubated in the acceptor bath solution to examine the changes of aortic tension. In addition, we examined effects of high temperature on the substances released from PVAT by heating (70°C) the transfer solution for 5 min that could damage the structure of peptide.

### Preparation of aortic homogenates

After incubated with PVAT for 15 min in the presence or absence of inhibitors, aortas were frozen with liquid nitrogen immediately and stored at −80°C before assay. Aortas were homogenized in 1× RIPA lysis buffer with protease inhibitors and phosphatase inhibitor at 4°C for 1.5 h. The aortic homogenates were then centrifuged at 4°C for 30 min at 16,000 *g*. The homogenates were collected and transferred to new centrifuge tubes to determine the protein concentrations by BCA protein assay kit.

### Western blot analysis

Each sample of aortic homogenates (including 100 µg proteins) was separated by 10% SDS-PAGE gel for 110 min, and then transferred to nitrocellulose membrane. The membranes were reacted with anti-eNOS (1∶500), anti-p-eNOS^Thr495^ (1∶200), anti-p-eNOS^Ser1177^ (1∶500), anti-AMPK (1∶500), anti-Cav-1 (1∶3000), and anti-*β*-actin (1∶10000) in a cold room overnight. Thereafter, membranes were washed and then reacted with secondary antibodies HRP conjugated goat anti-rabbit IgG or HRP conjugated goat anti-mouse IgG at room temperature for 2 h. After washed, the proteins were detected by Pierce ECL Western Blotting Substrate. Protein expressions were quantified by ImageJ software version 1.46r (National Institutes of Health, Bethesda, MD, USA).

### NO measurement

Samples were composed of 2 µl aortic homogenates and 4 µl 95% ice alcohol. The nitrite/nitrate concentrations in all samples were measured by using chemiluminescence through a NO analyzer (Sievers 280 Sievers Instruments, Inc., Boulder, CO, USA). It was noted that the aortic NO concentration depicted in the study was actually the total nitrite and nitrate concentrations in aortas. With this method, nitrate is reduced to NO via nitrite.

### Statistical analysis

All data were analyzed with Graphpad Prism software version 5.0 (Graphpad Prism software, Inc., La Jolla, CA, USA). All values were expressed as the mean ± SEM. The contractile response to PE was expressed as percentage of the maximal contraction induced by KCl (60 mM). Results of isometric tension studies were analyzed by two-way ANOVA analysis. Protein expressions were expressed as a ratio of the optical density for each protein against *β*-actin or eNOS. Both Protein expressions and nitrate/nitrite levels were analyzed with unpaired Student's *t*-test. Differences were considered statistically significant at *P*<0.05.

## Results

### PVAT-induced vasocontraction in aortas

The PE-induced vasocontraction was in a concentration-dependent manner in aortic rings with or without PVAT. In the presence of PVAT, the concentration-dependent vasocontraction induced by PE was significantly enhanced (*P*<0.05, Fig. 2A and 2B). Krebs' solution transfer did not influence the vasocontraction induced by PE (Fig. 2C and 2D).

### Effects of PVAT on aortic endothelial function

After denuding the endothelium (-E), the enhanced PE-induced vasocontraction by PVAT was inhibited (Fig. 2E and 2F). Similarly, in the presence of L-NAME (100 µM, an inhibitor of NOS via inhibiting NO production in the endothelium in our aorta preparations), the enhanced PE-induced vasocontraction by PVAT was also abolished (Fig. 2G and 2H). Furthermore, we assessed the modulating effect of PVAT on vasodilatation induced by ACh and SNP. Our results showed that PVAT significantly attenuated the ACh-induced vasodilatation (*P*<0.05, Fig. 2I and 2J), and increased the EC_50_ of ACh from 65.6±6.7 nM to 164.9±38.7 nM (*P*<0.05). We also observed that PVAT had no significant effect on SNP-induced vasodilatation (Fig. 2K and 2L). These results suggest that inhibition of NO in the endothelium plays an important role in enhanced vasocontraction induced by PVAT.

### Inhibitory effect of PVAT on vasodilatation induced by NO

**Figure 3:**
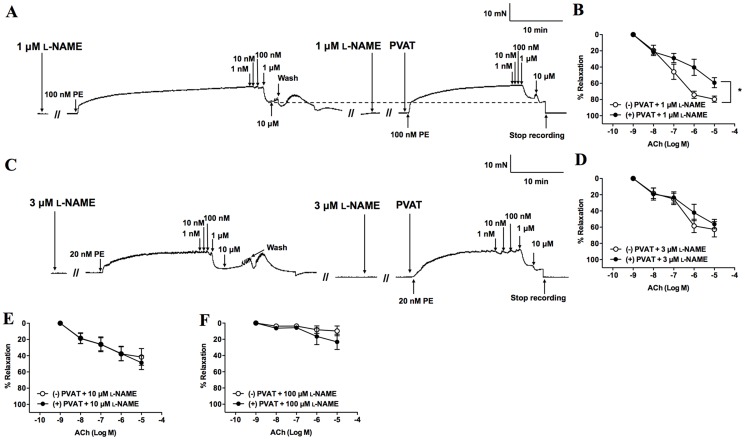
Inhibitory effects of perivascular adipose tissue (PVAT) on acetylcholine (ACh)-induced vasodilatation in aortas with various concentrations of N^ω^-nitro-L-arginine methyl ester (L-NAME). Original traces demonstrate dilator responses to incremental concentrations of ACh (1 nM∼10 µM) in the presence and absence of PVAT after pretreatment aortas with L-NAME (1 (A) or 3 µM (C)). After pretreatment of aortas with 1 µM L-NAME (n = 5) (B), 3 µM L-NAME (n = 5) (D), 10 µM L-NAME (n = 6) (E), or 100 µM L-NAME (n = 4) (F), vasodilator responses to ACh were performed in the presence and absence of PVAT. Values are means ± SEM for the numbers of animals in parentheses. *P<0.05 by two-way ANOVA compared with (−) PVAT group of same treatment group. (−) PVAT, absence of PVAT; (+) PVAT, presence of PVAT.

In order to evaluate whether PVAT could inhibit NO activity to enhance vasocontraction, aortic rings were incubated with different concentrations of L-NAME before carrying out the concentration-dependent ACh-induced vasodilatation. The inhibitory effect of PVAT on the ACh-induced vasodilatation was not affected by 1 µM L-NAME pretreatment (*P*<0.05, Fig. 3A and 3B). However, this inhibitory effect of PVAT on the ACh-induced vasodilatation was abolished by increasing the concentration of L-NAME up to 3, 10, and 100 µM (Fig. 3C - 3F).

### The contributions of PVAT-releasing factors or endothelial regulators in PVAT-induced vasocontraction

**Figure 4:**
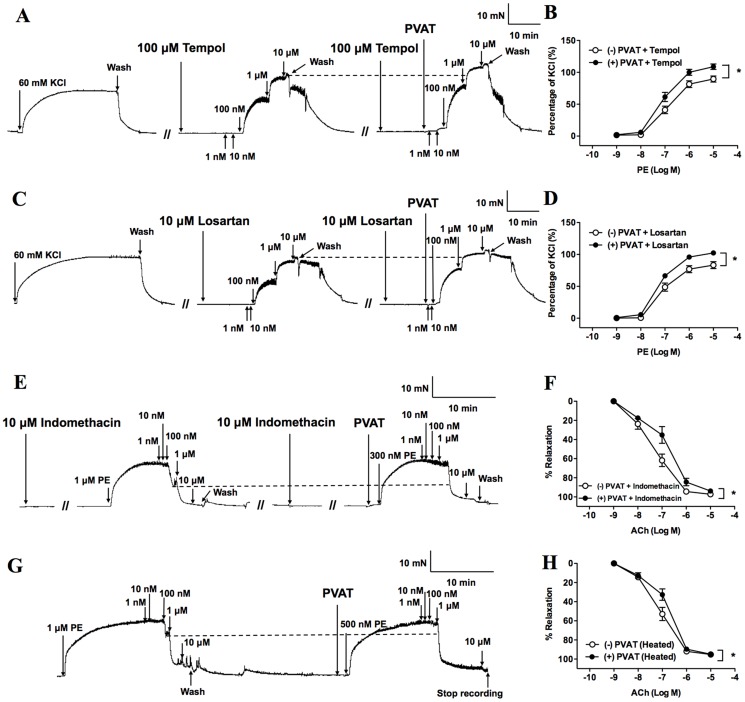
Effects of superoxide, angiotensin II, prostacyclin, and endothelin-1 on aortas in the presence and absence of perivascular adipose tissue (PVAT). Original traces demonstrate contractile responses to incremental concentrations of phenylephrine (PE) (1 nM∼10 µM) in the presence and absence of PVAT after pretreatment of aortas with 4-Hydroxy-TEMPO (tempol, 100 µM) (A) or losartan (10 µM) (C). After pretreatment of aortas with tempol (100 µM, n = 10) (B) or losartan (10 µM, n = 4) (D), concentration-response curves to PE were examined in the presence and absence of PVAT. Original traces demonstrate dilator response to incremental concentrations of ACh (1 nM∼10 µM) in the presence and absence of PVAT after pretreatment of aortas with indomethacin (10 µM) (E) or heating transfer solution of PVAT (G). After pretreatment aorta with indomethacin (10 µM, n = 7) (F) or heating transfer solution of PVAT to 70°C for 5 min (n = 6) (H), vasodilatation responses to ACh were examined in the presence and absence of PVAT. Values are means ± SEM for the numbers of animals in parentheses. *P<0.05 by two-way ANOVA compared with (−) PVAT group of same treatment group. KCl, potassium chloride; (−) PVAT, absence of PVAT; (+) PVAT, presence of PVAT.

In contrast to other evidences, the vasocontraction effects of PVAT were not significantly changed after pretreatment of aortas with tempol (a superoxide anion scavenger, 100 µM) (*P*<0.05, Fig. 4A and 4B) or losartan (an Ang II type 1 receptor antagonist, 10 µM) (*P*<0.05, Fig. 4C and 4D). This suggests that the PVAT-induced vasocontraction is not mediated by superoxide anion and Ang II in the current study. Since prostacyclin (PGI_2_) and endothelin-1 (ET-1) are known to be major vasoactive substances in the endothelium of thoracic aorta, we treated aortas with indomethacin (an inhibitor of COX, 10 µM) (*P*<0.05, Fig. 4E and 4F) or damaged the peptide structure of ET-1 by heating the PVAT-incubated solution (70°C for 5 min) (*P*<0.05, Fig. 4G and 4H). However, the inhibitory effect of PVAT on the ACh-induced vasodilatation was not changed. Thus, PVAT-enhanced vasocontraction was not caused by PGI_2_ or through ET-1 from the endothelium.

### Effects of PVAT on eNOS expression and activity

**Figure 5:**
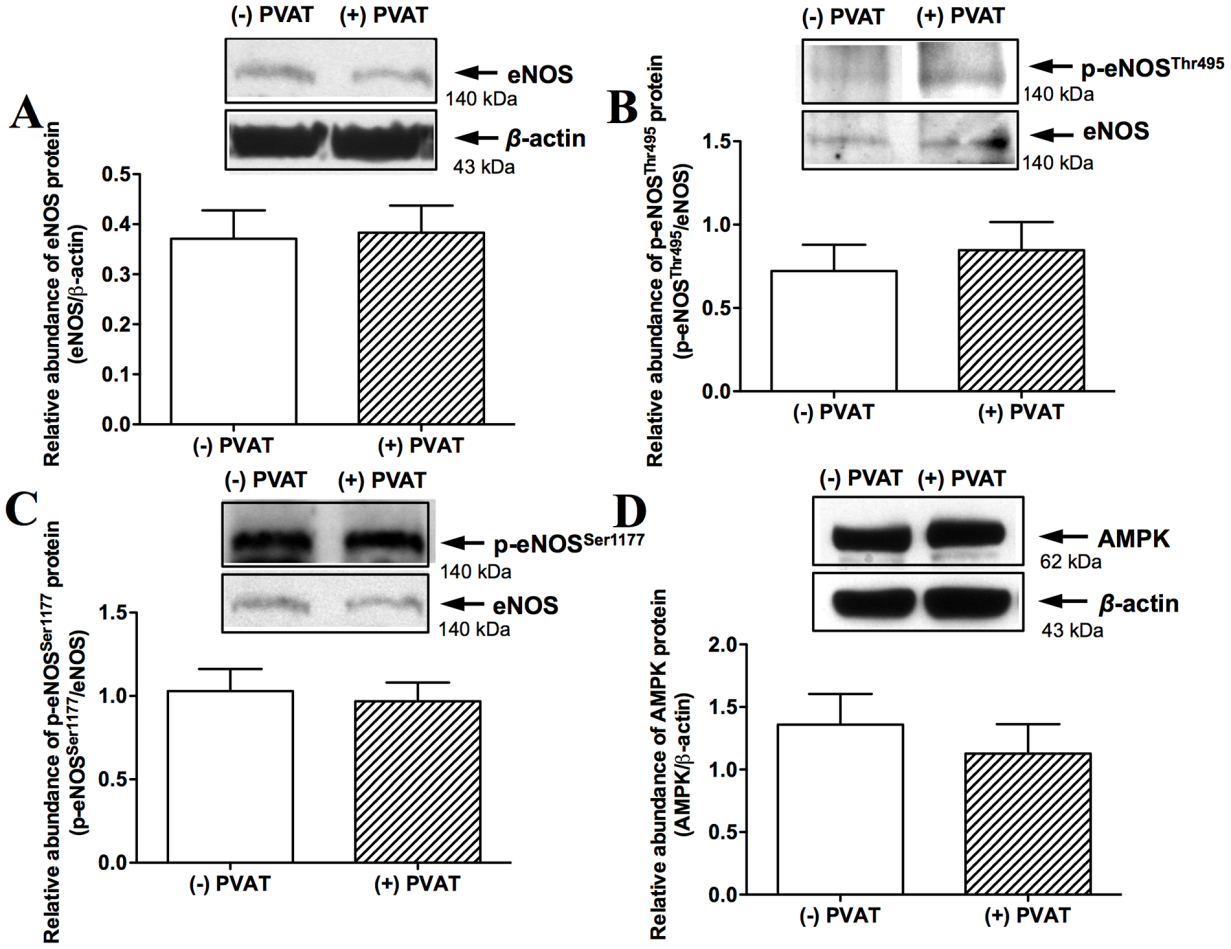
Effects of perivascular adipose tissue (PVAT) on endothelial nitric oxide synthase (eNOS) expression and phosphorylated residues. The aorta homogenates in the presence and absence of PVAT were used to measure eNOS protein expression (n = 26∼27) (A), eNOS phosphorylation on Thr^495^ (n = 7∼8) (B) or Ser^1177^ (n = 11) (C), and adenosine monophosphate-activated protein kinase (AMPK) expression (n = 5) (D). Blots are representative of the numbers of animals in parentheses. Densitometric mean values were normalized to *β*-actin or eNOS protein levels. (−) PVAT, absence of PVAT; (+) PVAT, presence of PVAT.

Our results showed that the protein expressions of eNOS (Fig. 5A) and two phosphorylated residues (Thr^495^ and Ser^1177^) were not changed after aortas incubated with PVAT transfer (Fig. 5B and 5C). In addition, there was no significant difference in AMPK (a modulator of eNOS activity) protein expression in aortas between with and without PVAT transfer (Fig. 5D). Thus, we suggest that the inhibitory effect of PVAT on vascular dilatation is not through modulating the expression or activity of eNOS.

### Role of Cav-1 in enhanced contraction caused by PVAT

**Figure 6:**
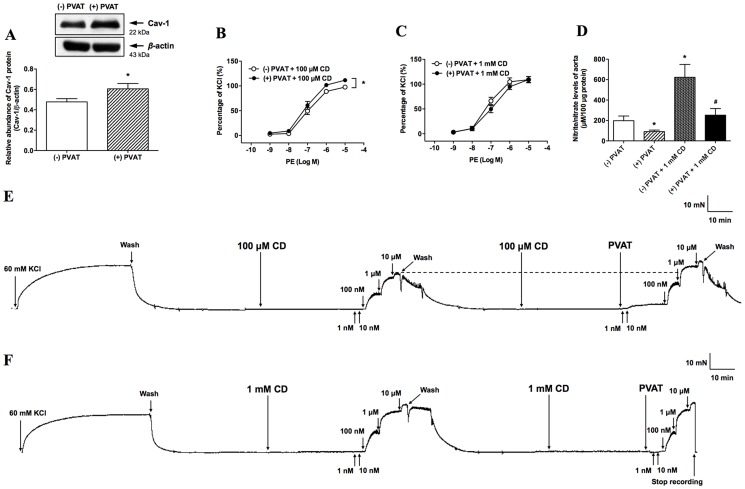
Role of caveolin-1 (Cav-1) in perivascular adipose tissue (PVAT)-enhanced vasocontraction. Western blot analysis of Cav-1 was performed in aorta homogenates in the presence (n = 11) and absence (n = 12) of PVAT (A). Blots are representative of the numbers of animals in parentheses. Densitometric mean values were normalized to *β*-actin protein levels. After aortas were preincubated with or without 100 µM methyl-*β*-cyclodextrin (CD) (n = 10) (B) or 1 mM CD (n = 11) (C), the phenylephrine (PE)-induced vasoconstriction was examined. Nitrate/nitrite levels were measured in aortic homogenates in the presence (n = 13) and absence (n = 12) of PVAT, and the addition of CD (1 mM) reversed the decreased nitrate/nitrite levels caused by PVAT (n = 3) (D). An original trace demonstrates contractile responses to incremental concentrations of PE (1 nM∼10 µM) in the presence and absence of PVAT after pretreatment aorta with CD (100 µM) (E) or 1 mM (F)). Values are means ± SEM for the numbers of animals in parentheses. *P<0.05, compared with (−) PVAT; ^#^ *P*<0.05, (+) PVAT+1 mM CD vs. (+) PVAT by unpaired Student's *t*-test. KCl, potassium chloride; (−) PVAT, absence of PVAT; (+) PVAT, presence of PVAT.

It has been shown that Cav-1 can occupy the Ca^2+^/CaM binding site of eNOS to inhibit eNOS activity [13]. Interestingly, the protein expression of Cav-1 was significantly increased in the presence of PVAT (*P*<0.05, Fig. 6A). The enhanced contractile effect of PVAT on the PE-induced vasocontraction was not affected by 100 µM CD pretreatment (*P*<0.05, Fig. 6B and 6E). However, this contractile effect of PVAT on the PE-induced vasocontraction was abolished by increasing the concentration of CD up to 1 mM (Fig. 6C and 6F). In addition, we found that the nitrate/nitrite levels were significantly decreased after PVAT treatment (*P*<0.05, Fig. 6D), which was reversed by the pretreatment of CD (1 mM) (*P*<0.05, Fig. 6D).

## Discussion

This study presented an inhibitory mechanism of PVAT on aortic endothelial function and we found that Cav-1, a negative regulator of eNOS, played an important role in PVAT-induced abnormal vascular responsiveness, i.e. enhanced vasoconstriction and impaired endothelial vasodilatation.

Adipose tissue is known to release numerous adipokines, and plays a paracrine role toward surroundings [16]. In 2002, Lò `hn *et al.* demonstrate that the anti-contractile effect of PVAT is mediated by a transferable factor, called ADRF [7]. Since that, the regulation of arterial tone by various adipokines has been confirmed [4], [17]. The adipokines with (i) both vasocontractile and vasorelaxing properties include reactive oxygen species [18], leptin [19], TNF-*α* [20], IL-6, and apelin [16]; (ii) vasocontractile adipokines include Ang II [21] and resistin; (iii) vasorelaxing adipokines include adiponectin, omentin, visfatin, and ADRF. In our study, we also observed the transferable characteristic of PVAT, which enhanced PE-induced contraction in rat thoracic aortas. This observation was similar to Gao and colleagues' finding that PVAT enhances the mesenteric artery contractile response to perivascular nerve stimulation [8]. They consider that the increased contraction is due to the production of superoxide induced by PVAT. However, our study showed that the enhanced PE-induced contraction by PVAT remained unchanged even after inhibition of superoxide anions and Ang II. The opposing results could be due to different methods of these studies used: (i) mesenteric arteries of Wistar-Kyoto rats, (ii) an intact connection between PVAT and the blood vessels, and (iii) electrical field stimulation to elicit vascular contractile response in isometric tension studies. On the contrary, our observation was opposite of the point of ADRF supported by Löhn [7], Gao [22], and Lee [23]. In our current study, we modified Löhn's experiment methods by using the PVAT transfer solution only. The donor chamber in Löhn's (they also showed a tracing about transfer of solution from isolated PVAT evoked vasodilatation; however, the PVAT-induced vasodilatation became not obviously compared with transfer of solution from intact aortic preparation) [7] or Gao's [22] experiments were composed of both PVAT and vessels. In order to eliminate the effects derived from vascular smooth muscle and endothelium from the donor, we used solution with PVAT alone. We found that PVAT-induced vasocontraction and anti-vasodilatation effects in rat thoracic aorta which were different form Lee's (vasorelaxation of PVAT on PE-induced contractions of rat thoracic aortic rings) [23] and Owen's (no effect of PVAT on potassium chloride-induced contractions of rat thoracic aortic rings) [24] findings. Nevertheless, if the unknown factors released from PVAT caused vasodilatation, the anti-vasodilatation effect should not be observed in Li's [25] and Tune's studies [9], [10]. In addition, the PVAT transfer in our study was incubated for 30 minutes independently and then replaced to an acceptor chamber for another 15 minutes for isometric tension study. The unknown factor(s) released from PVAT in our study must have a long-lasting characteristic which was different from Lee's (superfused rat thoracic aorta with PVAT superfusion) [23] and Owen's (PVAT was incubated with rat thoracic aorta for 30 min) [24] groups (the released substances from PVAT in their studies could be predominately short-lasting). Moreover, Li and colleagues claimed that an intact connection between PVAT and the blood vessel was important for transfer of whatever substance involved in this messaging [25]. It has been shown on multiple occasions that the presence of intact PVAT reduces the contractility of arteries to agonists [6], [26], [27], [28], [29], [30], [31]. This has been shown on almost all arteries, ranging from the aorta [6], [26], [27], mesenteric arteries [25], [28], skeletal arteries [29], [30], and subcutaneous arteries [31]. The reduced sensitivity to NA was independent of membrane potential, indicating that surrounded PVAT posed a diffusion limitation [25]. However, this concept from Li's [25] group could not explain that there was no effect of PVAT on ACh-induced vasodilation of mice resistance artery [29]. Therefore, the effects of PVAT on vasoreactivity vary among different vascular beds. In addition, when PVAT conditioned fluid is transferred from one myograph bath to another [7], [22], [23], [31], [32], [33], there is a vasodilatation effect. In our experiments, we adopted a slightly different approach in that we used a transfer solution which was derived from PVAT only – i.e. without the presence of the attached artery [7], [23]. In summary, we speculate that the fundamental differences of PVAT function between other publications and this study were owing to (i) the different degradations of substances released from PVAT (short-lasting vs. long-lasting characteristics), (ii) the varied methodology (clean and intact vessels vs. solution transfer experiment), and (iii) the diverse subjects (different species or vessels).

Intriguingly, the PVAT-enhanced PE-induced contraction was abolished by denuding the endothelium in our study. Although the anatomic barrier existed in the endothelium, paracrine effects of adipokines in PVAT may cause endothelial dysfunction (decreased NO production), hypercoagulability, increased chemotaxis and adhesion of monocytes to the endothelium [34]. Therefore, we proposed that the enhanced PE-induced contraction by PVAT could be due to the inhibition of endothelial function. Indeed, the ACh-mediated vasodilatation was reduced after PVAT treatment, whereas SNP-mediated endothelium-independent vasodilatation remained unchanged in this study. This suggests that the inhibitory effect of PVAT on vasodilatation is dependent on the endothelium.

There are several substances from the endothelium involving the regulation of vascular tone, including NO, endothelium-derived hyperpolarizing factor (EDHF), PGI_2_, and ET-1 [35]. It is highly unlikely that EDHF contributes to the vasocontraction effect of PVAT because EDHF plays a major role in the resistance arteries, but not in conductive arteries, in response to vasoactive substances or physical stimuli [36], [37]. Since the enhanced contractile effect of PVAT remained unchanged after suppressing the production of PGI_2_ by indomethacin, PGI_2_ was excluded in this study. Furthermore, after heating PVAT transfer solution to destroy peptides (e.g. ET-1 and Ang II), the enhanced contractile effect of PVAT was not changed, and hence, peptides seemed not to participate in the enhanced contractile effect of PVAT. Interestingly, L-NAME concentration-dependently inhibited the inhibitory effect of PVAT on ACh-induced vasodilatation, indicating that PVAT-induced endothelial dysfunction is through the inhibition of NO/eNOS pathway.

It has been shown that eNOS is regulated by a very complex network of protein kinases (PKA, PKB and PKC), protein phosphatases 1 and 2, cofactors (tetrahydrobiopterin, flavin mononucleotide and nicotinamide adenine dinucleotide phosphate), protein-protein interactions, and subcellular localization [38]. They regulate the phosphorylate sites of eNOS, e.g., Thr^495^, Ser^1177^, Ser^633^, Ser^615^ and Ser^114^ residues [38]. In particular, the Ser^1177^ residue is thought to be the most critical regulatory site for eNOS activation [38]. In addition, Payne *et al*. has proved that PVAT impairs coronary endothelial NO production via a PKC-*β*-dependent, site-specific phosphorylation of eNOS at Thr^495^ [10]. Nevertheless, in our study, there was no significant difference in eNOS, p-eNOS^Thr495^ and p-eNOS^Ser1177^ protein expression after PVAT pretreatment. It has been shown that AMPK activates eNOS on phosphorylate site of Ser^633^ [39], however, we did not detect the increased protein expression of AMPK after PVAT treatment. As for the impact of Ser^615^ and Ser^114^ on eNOS activity is controversial so far [38], and hence, we did not measure expression of Ser^615^ and Ser^114^ in this study. According to our results, we suggest that the reduction of NO level is not due to an increase of protein expression or activity of eNOS.

Cav-1 is present in most terminally differentiated cells, in particular in endothelial cells, adipocytes, and type II pneumocytes [40]. Cav-1 can inhibit NO production by occupying the Ca^2+^/CaM binding site of eNOS [13]. However, the interaction between Ca^2+^/CaM binding and regulation of phosphorylation in eNOS is still unclear. In this study, we first discovered that PVAT could enhance Cav-1 protein expression. In addition, the administration of CD, a cholesterol depleter to disassemble caveolae, eliminated the enhanced contractile effect of PVAT and reversed the decreased aortic NO level induced by PVAT. This suggests that PVAT increases Cav-1 protein expression and attenuates endothelial NO production leading to enhancement of contraction in aortas.

The inhibition of NO production is regarded as a characteristic of endothelial dysfunction. It has been shown that the impairment of endothelial function was a risk factor for the development of atherosclerosis [41]. Moreover, perturbations in the NO signaling have been implicated in numerous disease processes ranging from hypertension, shock and inflammation. In addition, PVAT accumulation plays a much more important role in coronary risk than the other body fat distributions in non-obese men [42]. This raises the concern between PVAT, aorta and cardiovascular events now and in the future.

**Figure 7:**
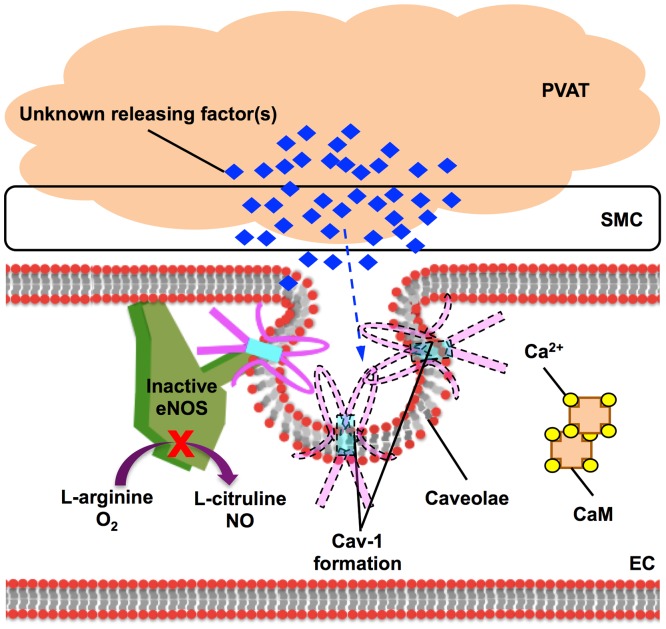
A proposed mechanism of perivascular adipose tissue (PVAT) in the regulation of vascular tone through the endothelium. We hypothesized that some unknown factor(s) released from PVAT entered to endothelium and caused caveolin-1 (Cav-1) formation. The Cav-1 probably competes the binding site on endothelial nitric oxide synthase (eNOS) with calcium/calmodulin (Ca^2+^/CaM), which results in eNOS inactivation and inhibition of nitric oxide (NO) production. The NO inhibition leads to enhanced vasoconstriction in rat aortas in isometric tension studies. SMC, smooth muscle cell; EC, endothelial cell.

In conclusion, our results provide direct evidence of PVAT elicited enhancement of vasoconstriction in rat aorta and it was endothelium-dependentvia increasing Cav-1 expression and decreasing NO production (Fig. 7). Therefore, our study reveals the connection of PVAT and endothelial dysfunction as an aspect in the future studies of cardiovascular diseases.
